# Synbiotic yogurt with nanoparticle entrapped rice straw hemicellulose for immediate probiotic support and prebiotic delivery

**DOI:** 10.1038/s41538-025-00539-z

**Published:** 2025-08-18

**Authors:** Shaymaa A. Ismail, Bahgat Fayed, Hoda S. El-Sayed, Hanan A. A. Taie, Amira A. Hassan

**Affiliations:** 1 Department of Chemistry of Natural and Microbial Products, Pharmaceutical and Drug Industries Research Institute, National Research Centre, 33 El Bohouth St., Dokki, 12622 Giza, Egypt; 2 Dairy Department, Food Industries and Nutrition Research Institute, National Research Centre, 33 El Bohouth St., Dokki, 12622 Giza, Egypt; 3 Plant Biochemistry Department, Agricultural and Biology Research Institute, National Research Centre, 33 El Bohouth St., Dokki, 12622 Giza, Egypt

**Keywords:** Biotechnology, Microbiology

## Abstract

This study presents a novel synbiotic yogurt fortified with free and PLGA-entrapped rice straw hemicellulose hydrolyzate (RSHH) to offer immediate probiotic support and sustained prebiotic delivery. RSHH, obtained by enzymatic hydrolysis, yielded 0.5 g/g hemicellulose and was rich in xylooligosaccharides (48.3% reducing sugars), with notable antioxidant activity (45.59 µmol Trolox/mg) and anticancer effects (61.98% reduction in EAC cell viability at 1000 µg/mL). It enhanced the growth of *Lactiplantibacillus paraplantarum* and *Bifidobacterium bifidum* more effectively than inulin. Biosafety tests in rats confirmed no adverse effects and improved lipid profiles. RSHH was successfully encapsulated into PLGA nanoparticles (38.77% efficiency, 204.9 nm size, –18.3 mV zeta potential), with spherical morphology, uniform distribution and no aggregation as confirmed by TEM. Yogurt containing both free and entrapped RSHH demonstrated the best performance over 20 days. This dual-delivery system offers a promising solution to enhance probiotic viability and prebiotic stability in functional food applications.

Synbiotics offer a promising strategy for improving human health. These formulations combine probiotics and prebiotics to enhance the host’s gut microbiota and overall well-being^1,2^. Probiotics, such as *Lactobacillus* and *Bifidobacterium* species, are live microorganisms that provide health benefits when consumed in sufficient quantities^3–5^. Prebiotics, on the other hand, are non-digestible food components, often fibers, that selectively stimulate the growth and activity of beneficial gut bacteria^4,6–10^.

The combined use of probiotics and prebiotics in synbiotics provides distinct advantages over their individual use. This synergistic interaction amplifies the effectiveness of both components^11^. Prebiotics serve as a nutrient source for probiotics, promoting their colonization and activity in the gut. This results in improved modulation of gut microbiota^6^. Hence, synbiotics offer a more robust and sustained enhancement of gut flora compared to using probiotics or prebiotics individually. They have demonstrated greater efficacy in treating various gastrointestinal conditions, including irritable bowel syndrome (IBS), inflammatory bowel disease (IBD), and antibiotic-associated diarrhea^12–14^. The dual approach of introducing beneficial bacteria and supporting their growth leads to better health outcomes. Furthermore, synbiotics are more effective at preventing the overgrowth of harmful bacteria by both introducing competitive probiotic strains and supporting their growth with prebiotics^2^. This dual action helps prevent infections and maintains a healthy balance of gut flora^15^.

Recent research also suggests that synbiotics may reduce the risk of colorectal cancer by modulating the gut microbiome and influencing metabolic and inflammatory pathways linked to cancer development^16^. In addition, synbiotics have been shown to strengthen the immune system by enhancing immune cell activity, increasing antimicrobial peptide production, and reducing inflammation, making them a potential option for managing autoimmune diseases and infections by regulating gut microbiota composition, and enhancing regulatory T cells^17–19^. They are also being explored for their beneficial effects on metabolic health, including improved insulin sensitivity, reduced lipid levels, and better energy metabolism, offering potential interventions for obesity, diabetes, and metabolic syndrome^20–22^. Recent studies indicate that synbiotics may positively impact skin health through the gut-skin axis, helping to manage conditions like acne, eczema, and psoriasis by reducing inflammation and oxidative stress^23^. Additionally, synbiotics are being investigated for their role in mental health by influencing the gut-brain axis, potentially alleviating conditions such as anxiety, depression, and cognitive decline through improved gut health and reduced systemic inflammation^1,9,24^.

Building on these promising findings, the application of synbiotics in yogurt and dairy products has attracted significant attention due to the natural compatibility of these products with probiotic and prebiotic ingredients. Yogurt and other fermented dairy products serve as an ideal medium for delivering beneficial bacteria, such as *Lactobacillus* and *Bifidobacterium* strains, along with prebiotic fibers like inulin or oligosaccharides^3,7^. The inclusion of synbiotics in these products enhances their health-promoting properties by not only providing live beneficial bacteria but also ensuring their growth and survival during digestion. Synbiotic-enriched dairy products have been linked to improve digestive health, immune system support, and potential benefits in metabolic conditions like obesity and diabetes^25^. Moreover, these products offer an accessible and convenient way for consumers to incorporate probiotics and prebiotics into their daily diet^3^.

One significant challenge in the development of synbiotic yogurt and other dairy products is that prebiotics may be consumed by the probiotic bacteria during storage. The probiotics in the yogurt may utilize these prebiotics as a nutrient source, leading to a depletion of prebiotics before the product is even consumed. This premature consumption of prebiotics can diminish their intended effects in the digestive system, reducing the synbiotic product’s health benefits. Entrapment of prebiotics into PLGA nanoparticles presents a promising solution to prevent the premature consumption of prebiotics by probiotic bacteria during the storage of synbiotic yogurt and dairy products^6^. PLGA is a biodegradable and biocompatible polymer widely used in drug delivery systems, and it can effectively entrap bioactive compounds, protecting them from the surrounding environment until they reach their target site^26–28^. When prebiotics are entrapped in PLGA nanoparticles, they are shielded from being metabolized by probiotics during storage. This entrapment ensures that the prebiotics remain intact until they are released in the gastrointestinal tract, where they can exert their beneficial effects. The controlled release properties of PLGA nanoparticles allow for a sustained and targeted delivery of prebiotics in the gut, maximizing their impact on gut microbiota and enhancing the overall efficacy of the synbiotic product. Hence, entrapping prebiotics in PLGA nanoparticles offers a novel and effective strategy to overcome the problem of premature prebiotic consumption in synbiotic dairy products, enhancing both their stability and functionality^29^.

Herein, prebiotic oligosaccharides-rich hydrolysate was produced through the enzymatic hydrolysis of rice straw hemicellulose. This prebiotic hydrolysate was then incorporated into yogurt, both as free prebiotics and entrapped within PLGA nanoparticles, alongside probiotic bacteria (Fig. 1). The novelty of this approach lies in its ability to balance the immediate needs of probiotics during storage with the long-term health benefits of prebiotics in the gut. By preserving a portion of the prebiotics for release in the intestine, this strategy enhances the synbiotic product’s effectiveness in modulating gut microbiota, improving digestion, and supporting overall health.

**Fig. 1:**
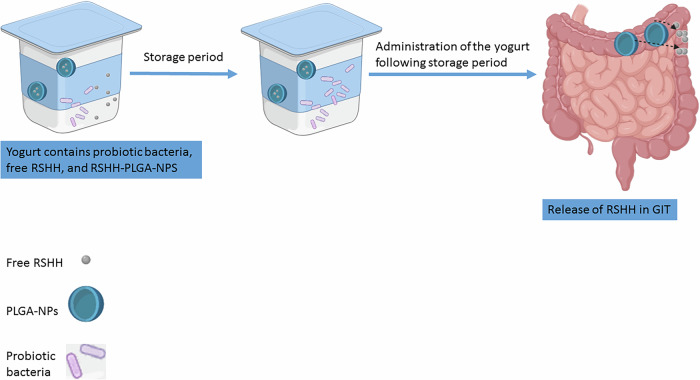
Schematic representation of the innovative synbiotic yogurt formulation. The yogurt contains probiotic bacteria, free rice straw hemicellulose hydrolyzate (RSHH), and RSHH encapsulated within PLGA nanoparticles (RSHH-PLGA-NPs). During storage period, free RSHH provides immediate probiotic support, while after the storage period, RSHH-PLGA-NPs will allow RSHH for the gastrointestinal tract (GIT) microbiota enrichment.

This study aims to develop and evaluate a novel dual-delivery synbiotic yogurt incorporating prebiotic oligosaccharides-rich hydrolysate from rice straw hemicellulose in both free and PLGA-encapsulated forms. The objective is to enhance probiotic viability during storage while ensuring a controlled release of prebiotics in the intestine, maximizing gut health benefits.

We hypothesize that incorporating free prebiotics will sustain probiotic viability during storage, while PLGA-encapsulated prebiotics will provide a controlled release in the intestine, leading to an improved modulation of gut microbiota compared to conventional synbiotic formulations.

## Results

### Production of bioactive RSHH from rice straw hemicellulose

Hemicellulose, a naturally occurring biopolymer, has been widely investigated for its applications in food, medicine, and energy industries^30^. In the current study, hemicellulose was extracted from rice straw using 4% NaOH at 80 °C for 1 h, followed by precipitation with ethanol. The process yielded approximately 1.6 g of hemicellulose per 10 g of rice straw.

The extracted hemicellulose was hydrolyzed enzymatically using xylanase from *Aspergillus terreus* with an enzyme-to-substrate ratio of 3.75 U/mg. The hydrolysis reaction yielded 0.5 g of RSHH per gram of hemicellulose. The produced RSHH was characterized by its physicochemical properties. The moisture content was 26 ± 1.4%, the total reducing sugar content was 48.3 ± 0.9%, reflecting a high concentration of oligosaccharides derived from the enzymatic hydrolysis of hemicellulose with the total phenolic content was 0.74 ± 0.01%. The ash content was found to be 23.9 ± 0.1%.

**Fig. 2:**
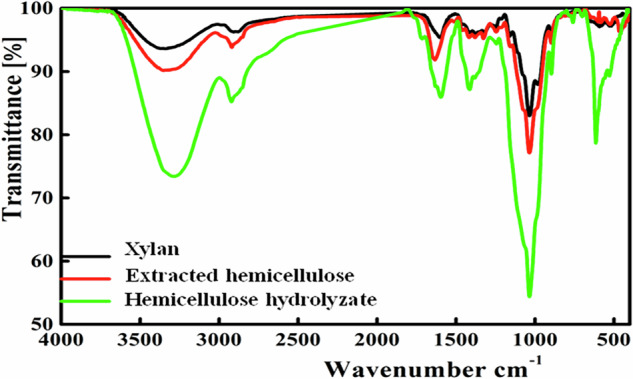
FTIR spectrum of RSHH compared with reference compounds (hemicellulose and commercial xylan). The spectrum highlights characteristic peaks, including OH stretching ( ~ 3300 cm^-1^), C–H stretching ( ~ 2900 cm^-1^), and β-glycosidic linkage ( ~ 900 cm^-1^).

FTIR analysis of the RSHH revealed characteristic peaks as shown in Fig. 2 aligning with extracted hemicellulose and commercial xylan: ~3300 cm^-1^ (OH stretching), ~2900 cm^-1^ (C–H stretching), ~1600 cm^-1^ (bending of water molecules), ~1400 cm^-1^ (C-H, C-OH, or C–O bond bending), ~1030 cm^-1^ (C–OH, C–O, or C–C bond stretching), ~900 cm^-1^ (β-glycosidic linkage), ~620 cm^-1^ (C–C–H stretching), and ~500 cm^-1^ (C–O–C bending). Previously, we analyzed RSHH by HPLC in another study and found that the oligosaccharides primarily consisted of xylooligosaccharides, including xylobiose (240.68 mg/g) and xylotriose (17.79 mg/g), as the main products. Additionally, the monosaccharides detected were xylose (146.73 mg/g), arabinose (54.95 mg/g), and glucose (36.43 mg/g)^31^.

These results demonstrate the effective conversion of rice straw hemicellulose into value-added hydrolyzate, which holds potential for applications in prebiotic formulations and functional foods.

### Bioactivity of RSHH

**Fig. 3:**
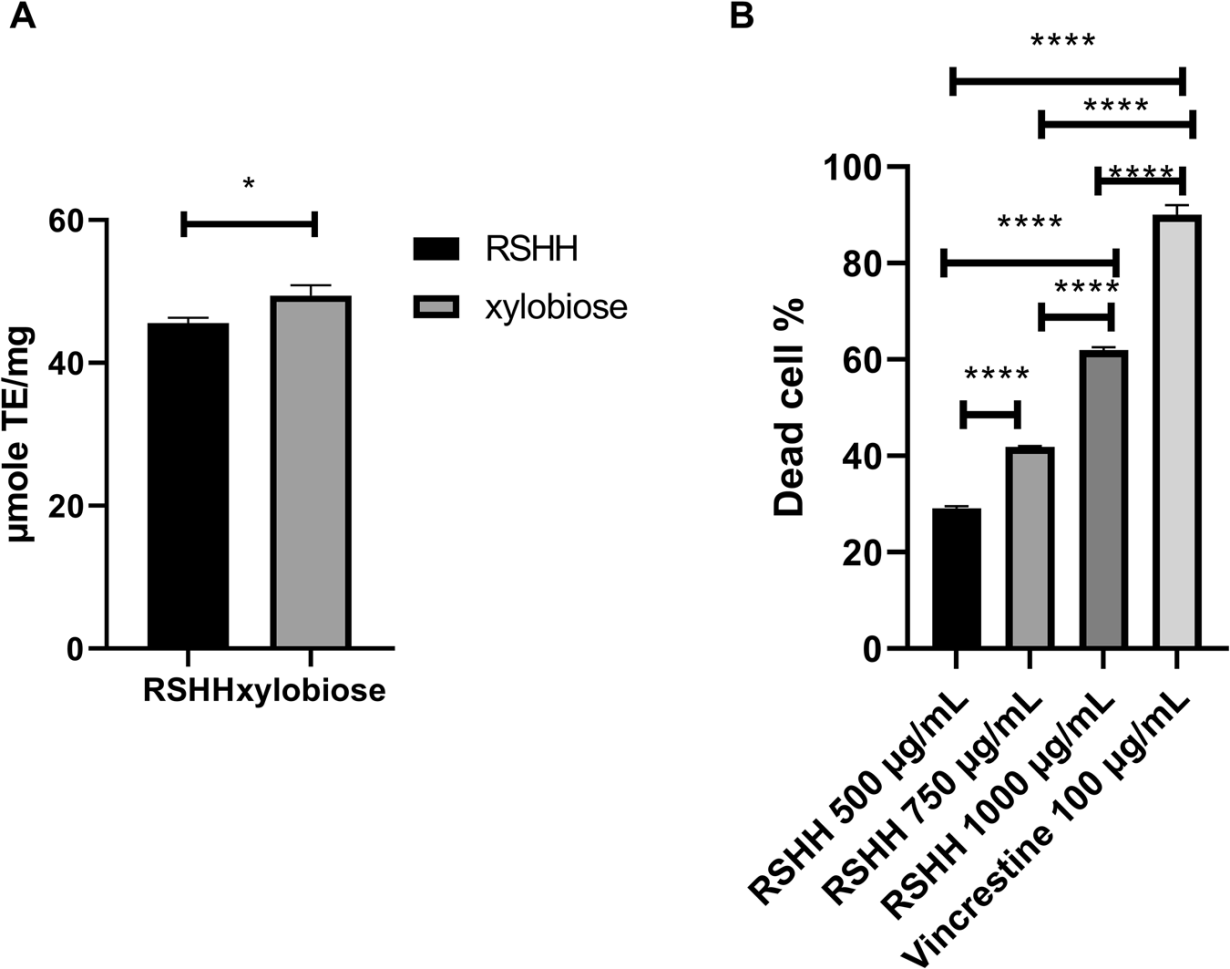
Bioactivity of RSHH. **A** Antioxidant activity of RSHH compared to standard xylobiose as measured by DPPH radical scavenging assay. **B** Cytotoxic effect of RSHH on EAC cells at different concentrations.

In the current study, the antioxidant activity of the RSHH was compared with that of standard xylobiose and xylotriose by determining its efficiency in scavenging DPPH radicals (Fig. 3A). The results indicated that the RSHH sample possessed an antioxidant activity of 45.59 ± 0.75 µmole TE/mg, while the standard xylobiose exhibited an antioxidant activity of 49.42 ± 1.44 µmole TE/mg. No antioxidant activity was detected for xylotriose.

Further, as illustrated in Fig. 3B, the RSHH exhibited a concentration-dependent inhibition of EAC cell viability. At a concentration of 1000 µg/mL, the RSHH achieved the highest inhibition rate, reducing cell viability by 61.98 ± 0.59%. This was followed by a reduction of 41.85 ± 0.20% at 750 µg/mL, while the lowest effect was observed at 500 µg/mL, with a reduction of 29.10 ± 0.44%.

Regarding the prebiotic activity, Table 1 presents the growth behavior of probiotic strains when 2% of the RSHH sample, xylose, and inulin were added as prebiotic agents, compared to MRS broth containing glucose as a carbohydrate growth supporter ( + ve control). All prebiotic treatments (RSHH, inulin, and xylose) significantly enhanced the growth of the investigated strains above their initial counts (*P* = 0.0001, 0.0001, and 0.0002, respectively). The sugar levels provided by the prebiotic treatments led to a greater increase in bacterial numbers compared to MRS broth, with significant differences observed only for RSHH (*P* = 0.0013, 0.102, and 0.150 for RSHH, inulin, and xylose, respectively. Notably, the RSHH sample demonstrated a significantly greater ability to enhance the viability of the tested probiotic strains (*L. rhamosus*, *L. paraplantarum*, *L. Salivarius*, *L. Gessarii*, *B. Lactis*, *B. bifidum*) compared to media enriched with other commercial prebiotics, such as inulin (*P* = 0.0087). This highlights the RSHH’s superior prebiotic activity and its stronger influence on probiotic strain development compared to traditional prebiotics like inulin. The effect of the RSHH sample on pathogenic strains was also evaluated. Table 2 indicates that none of the tested prebiotics, including the RSHH sample, showed any notable activation of pathogen growth compared to the positive control (*P* >0.05). For instance, *S. aureus* growth increased from an initial 3.45 ± 0.021 log CFU/mL to 5.69 ± 0.03 log CFU/mL in the positive control, whereas in the presence of RSHH, it reached only 4.60 ± 0.035 log CFU/mL. Similarly, *E. coli* increased from 3.80 ± 0.01 log CFU/mL to 5.52 ± 0.033 log CFU/mL in the positive control but remained lower at 4.95 ± 0.022 log CFU/mL with RSHH. This pattern was consistent across all tested pathogens, confirming that RSHH did not significantly promote their growth. Pathogen counts in the nutrient broth containing the RSHH remained within the same log cycle as those in the positive control or the uninoculated nutrient medium. This result confirms that the RSHH sample selectively supports the growth of beneficial probiotic strains without promoting the proliferation of harmful pathogens.

**Table 1:** Effect of different prebiotic agents on the growth of probiotic strains (log CFU/mL)

Probiotic/concentration %	*Lb. Rhamosus*	*Lb. paraplantarum*	*Lb. Salivarius*	*Lb. Gessarii*	*Bif. Lactis*	*Bif. bifidum*
**Initial Inoculation counts**	8.00 ± 0.035	7.88 ± 0.030	8.49 ± 0.028	7.96 ± 0.025	8.00 ± 0.03	8.15 ± 0.033
**MRS**	9.10 ± 0.046	10.07 ± 0.035	10.40 ± 0.040	9.23 ± 0.028	9.80 ± 0.033	9.95 ± 0.045
**RSHH sample (2%)**	11.20 ± 0.025	11.34 ± 0.030	11.50 ± 0.040	10.69 ± 0.033	11.00 ± 0.045	10.56 ± 0.04
**Xylose (2%)**	10.56 ± 0.050	10.43 ± 0.038	10.90 ± 0.044	9.70 ± 0.04	10.35 ± 0.051	9.33 ± 0.03
**Inulin (2%)**	10.90 ± 0.033	11.15 ± 0.050	11.00 ± 0.045	9.75 ± 0.03	10.17 ± 0.035	9.40 ± 0.028

Values are expressed as mean ± standard deviation (SD) of three independent experiments. Higher values indicate better prebiotic potential.

**Table 2:** Pathogen growth in the presence of different prebiotics (log CFU/mL)

Probiotic/concentration %	*S. aureus*	*E. coil*	*L. monocytogenes*	*S. Typhamirum*	*A. flaus*	*C. albicans*
**Initial inoculation counts**	3.45 ± 0.021	3.80 ± 0.01	3.40 ± 0.03	3.50 ± 0.02	3.22 ± 0.032	3.39 ± 0.02
**Positive Control**	5.69 ± 0.03	5.52 ± 0.033	5.62 ± 0.029	5.31 ± 0.038	5.10 ± 0.03	5.37 ± 0.025
**RSHH sample**	4.60 ± 0.035	4.95 ± 0.022	4.82 ± 0.03	5.00 ± 0.027	4.75 ± 0.025	5.24 ± 0.03
**Xylose**	5.12 ± 0.031	5.33 ± 0.035	4.76 ± 0.029	5.29 ± 0.03	5.30 ± 0.01	5.33 ± 0.029
**Inulin**	5.18 ± 0.021	5.69 ± 0.03	5.00 ± 0.027	5.35 ± 0.031	5.80 ± 0.025	5.40 ± 0.018

Values are mean ± SD from three replicates. The positive control represents growth in nutrient broth without prebiotic addition. Lower values indicate better pathogen inhibition.

### In-vivo biosafety

**Fig. 4:**
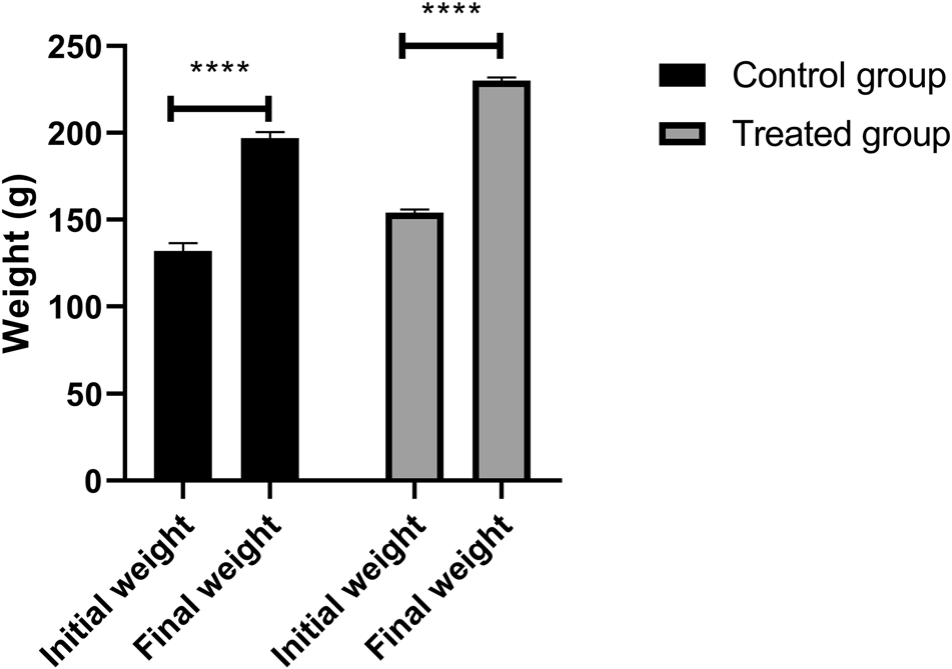
Effect of RSHH administration on the body weight of Wistar rats.

Rats were weighed on days 0, 14, and 28 of the experiment. The results showed a gradual increase in body weight in both the control and treated groups, following a similar pattern (Fig. 4). The initial weight of the control group was 132 ± 4.51 g, while that of the treated group was 154 ± 2.00 g. By the end of the experiment, the control group reached 197 ± 3.61 g, and the treated group reached 230 ± 2.00 g, reflecting a 49.24% and 49.35% weight increase for the control and treated groups, respectively. Table 3 indicates normal values in organ weights after administering the RSHH. However, Table 4 demonstrates that the administration of RSHH improved the lipid profile in the treated animals compared to the control group. Total cholesterol, triglycerides, and LDL cholesterol levels in the treated group slightly decreased (not significantly) to 134 ± 1.07, 93 ± 0.94, and 36.0 ± 0.49 mg/dL, respectively, compared to 140 ± 0.16, 99 ± 0.97, and 36.0 ± 0.49 mg/dL in the control group. In contrast, HDL cholesterol increased significantly (*P* = 0.0001) to 64.2 ± 0.61 mg/dl in the treated group compared to 53.7 ± 0.28 mg/dl in the control group, reflecting an overall improvement in the lipid profile. Interestingly, Table 5 confirms that liver function markers (ALT and AST) in the treated group did not show significant changes (*P* > 0.05) compared to the control group at 14 or 28 days. Meanwhile, supplementation with RSHH for four weeks reduced blood creatinine and urea levels to 0.37 ± 0.02 and 5.13 ± 0.05 mg/dl, respectively, compared to 0.45 ± 0.02 and 6.73 ± 0.13 mg/dl in the control group (*P* = 0.0001). Although serum glucose levels increased significantly to 111 ± 1.53 mg/dl in the treated group (*P* = 0.0001), they remained within normal ranges.

**Table 3:** Effect of RSHH administration on organ weights in male wistar rats (g)

Samples	Brain	Liver	Kidney	Spleen	Lungs	Testes	Heart
**Control**	1.27 ± 0.09	6.81 ± 0.19	1.57 ± 0.23	0.698 ± 0.03	1.41 ± 0.054	3.92 ± 0.06	0.623 ± 0.04
**RSHH**	1.43 ± 0.06	6.92 ± 0.04	1.80 ± 0.29	0.835 ± 0.09	1.45 ± 0.045	4.02 ± 0.158	0.644 ± 0.02

Results are expressed as Mean ± SD for six animals per group.

**Table 4:** Effect of administration of RSHH on lipid profile in the serum of male wistar rats

Samples	Lipid Profile							
	T. Cholesterol(mg/dl)		HDL-C (mg/dl)		LDL-C (mg/dl)		Triglyceride (mg/dl)	
	days		days		Days		days	
	14	28	14	28	14	28	14	28
Control	140 ± 0.43	140 ± 0.16	53.7 ± 0.31	53.7 ± 0.28	40.6 ± 0.25	40.6 ± 0.44	99 ± 0.06	99 ± 0.97
RSHH	138 ± 0.10	134 ± 1.07	58.4 ± 0.19	64.2 ± 0.61	38.4 ± 0.27	36.0 ± 0.49	97 ± 0.19	93 ± 0.94

Results are expressed as Mean ± SD for six animals per group.

**Table 5:** Effect of administration of RSHH on liver, kidney functions and serum glucose of male Wistar Rats

Samples	Liver function				Kidney function				Serum glucose	
	ALT (U/L)		AST (U/L)		Urea (mg/dl)		Creatinine (mg/dl)		(mg/dl)	
	Days									
	14	28	14	28	14	28	14	28	14	28
Control	33.4 ± 0.39	33.4 ± 0.37	130 ± 0.19	129 ± 0.45	6.90 ± 0.08	6.73 ± 0.13	0.46 ± 0.02	0.45 ± 0.02	97 ± 2.00	99 ± 0.58
RSHH	33.7 ± 0.22	32.5 ± 0.30	129 ± 0.56	129 ± 0.37	6.68 ± 0.14	5.13 ± 0.05	0.45 ± 0.01	0.37 ± 0.02	103 ± 1.00	111 ± 1.53

Results are expressed as Mean ± SD for six animals per group.

### Characterization of PLGA nanoparticles loaded with RSHH

**Fig. 5:**
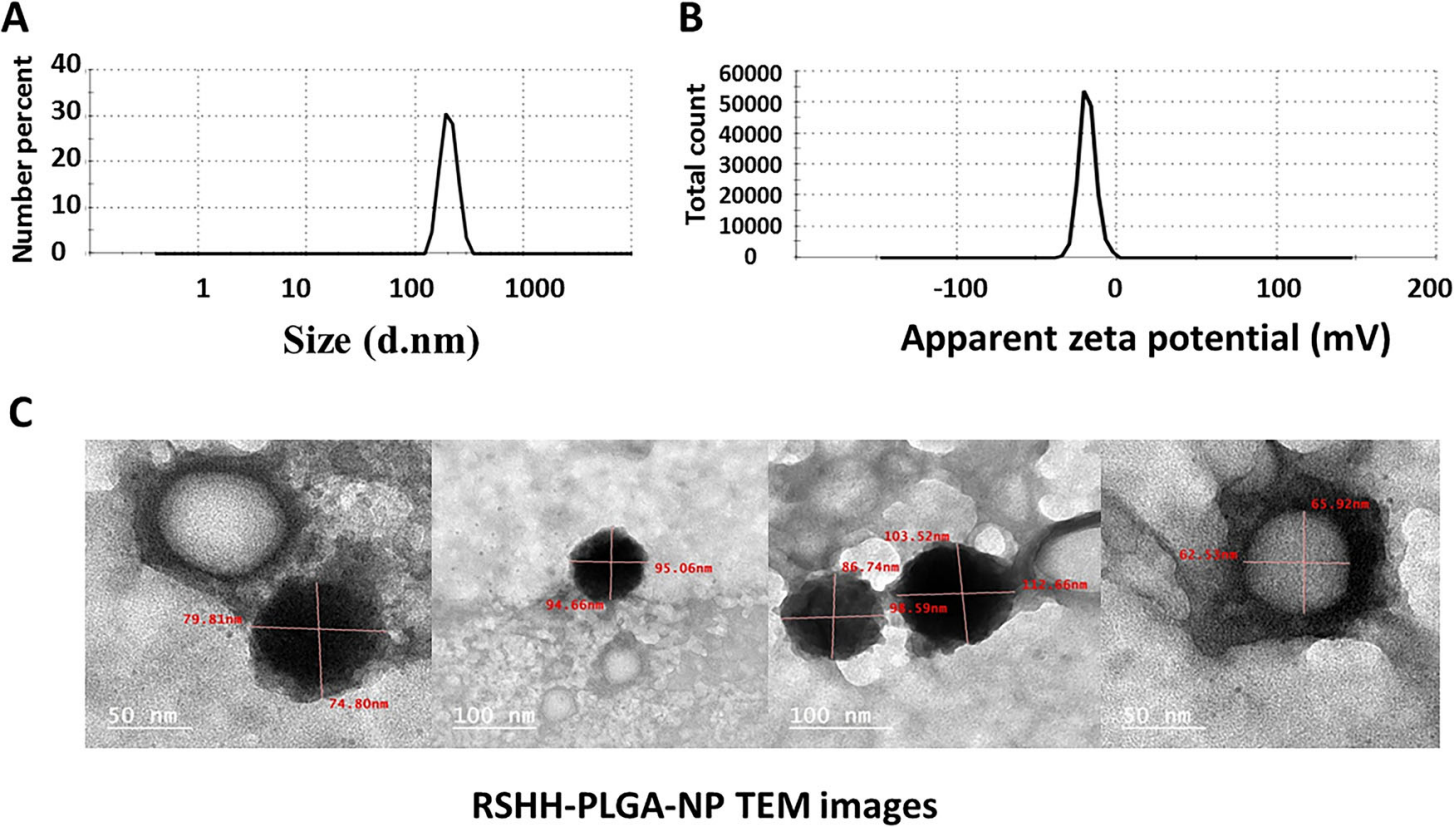
Characterization of RSHH-loaded PLGA nanoparticles (RSHH-PLGA-NP). **A** Particle size distribution, **B** Zeta potential analysis, and **C** TEM images depicting the spherical morphology and uniform dispersion of nanoparticles, taken at different magnifications to highlight structural integrity.

The entrapment efficiency of RSHH within PLGA nanoparticles was calculated indirectly and determined to be 38.77%. The particle size, measured using a zeta sizer, showed an average size of 204.9 ± 35.77 nm, as illustrated in Fig. (5A). Additionally, the zeta potential was recorded as -18.3 ± 5.32 Mv (Fig. 5B). The particles were further characterized by TEM, which revealed that they were spherical in shape, free from aggregation, and had a size consistent with the measurements obtained from the zeta sizer, as shown in Fig. (5C).

### Stirred Yogurt Evaluation

The count of the loaded probiotic *L. paraplantarum* in stirred yogurt samples was measured during storage periods, as shown in Table 6. All samples exhibited an increase in bacterial counts within the first 15 days. The sample fortified with free RSHH exhibited the highest bacterial count at 10.00 log CFU/g, followed by the sample containing both free and RSHH-PLGA-NP (9.88 log CFU/g). The sample containing only RSHH-PLGA-NP maintained a lower but stable bacterial count of 8.90 log CFU/g at 15 days. Notably, the *L. paraplantarum* count at 15 days for the free RSHH sample was significantly different from that of the RSHH-PLGA-NP group (*P* = 0.0001). However, no significant difference was observed when compared to the combined treatment of free RSHH and RSHH-PLGA-NP (*P* = 0.086). By day 20, the counts began to decline more significantly in all the samples.

**Table 6:** Counts of *L. paraplantarum* counts of stirred yogurt during cold storage periods (log CFU/mL)

Storage period (days)	Control	Free RSHH sample	RSHH-PLGA-NP sample	Free RSHH + RSHH-PLGA-NP
**Fresh**	7.18 ± 0.028	7.90 ± 0.03	7.20 ± 0.022	7.82 ± 0.031
**5**	7.94 ± 0.018	8.85 ± 0.02	8.10 ± 0.025	8.80 ± 0.03
**10**	8.18 ± 0.021	9.50 ± 0.022	8.65 ± 0.018	9.10 ± 0.015
**15**	8.33 ± 0.020	10.00 ± 0.03	8.90 ± 0.019	9.88 ± 0.09
**20**	8.00 ± 0.032	9.80 ± 0.025	8.18 ± 0.03	9.40 ± 0.011

Data expressed as a mean of three replicates.

The starter cultures (*S. thermophilus* and *L. bulgaricus*) counts were evaluated during the storage period, as presented in Table 7. Both cultures exhibited a gradual increase during the first 15 days across all treatments. The sample containing free RSHH had the highest counts for both cultures, followed by the sample containing both RSHH and RSHH-PLGA-NP. The RSHH-PLGA-NP and control samples exhibited lower counts. Statistically, *S. thermophilus* and *L. bulgaricus* counts showed no significant differences between the free RSHH and combined RSHH + RSHH-PLGA-NP treatments at day 15. However, RSHH-PLGA-NP exhibited a significant decline compared to free RSHH (*P* = 0.011 and *P* = 0.0001 for *S. thermophilus* and *L. bulgaricus*, respectively). By day 20, all samples showed a minimal decline in bacterial count.

**Table 7:** the stirred yogurt starter cultures during storage (log CFU/mL)

Storage periods (days)	Control	Free RSHH sample	RSHH-PLGA-NP	RSHH + RSHH-PLGA-NP
***Streptococcus thermophiles***				
**Fresh**	7.18 ± 0.10	7.80 ± 0.093	7.20 ± 0.044	7.60 ± 0.035
**5**	7.58 ± 0.04	8.50 ± 0.098	7.90 ± 0.048	8.00 ± 0.08
**10**	8.40 ± 0.03	9.35 ± 0.098	8.28 ± 0.031	9.00 ± 0.09
**15**	8.85 ± 0.038	9.75 ± 0.24	8.90 ± 0.05	9.42 ± 0.095
**20**	8.70 ± 0.033	9.50 ± 0.18	8.47 ± 0.03	9.11 ± 0.073
***Lactobacillus bulgaricus***				
**Fresh**	7.00 ± 0.040	7.72 ± 0.033	7.10 ± 0.03	7.45 ± 0.044
**5**	7.58 ± 0.035	8.33 ± 0.084	7.84 ± 0.027	8.00 ± 0.033
**10**	8.12 ± 0.03	9.48 ± 0.072	8.37 ± 0.035	9.30 ± 0.07
**15**	8.88 ± 0.027	9.94 ± 0.16	8.67 ± 0.021	9.70 ± 0.09
**20**	8.18 ± 0.033	9.54 ± 0.028	8.42 ± 0.011	9.55 ± 0.028

Data expressed as a mean of three replicates.

Further, the microbial strains’ activities during storage significantly impacted the pH levels of the stirred yogurt. Table 8 presents the results. Fresh samples recorded pH values ranging from 4.40 to 4.51. Over the storage period, the pH decreased gradually, reaching 4.10, 3.80, 4.22, and 3.92 for the control, free RSHH sugar, RSHH-PLGA-NP, and RSHH + RSHH-PLGA-NP, respectively, by the end of storage. Samples containing free RSHH and RSHH + RSHH-PLGA-NP exhibited lower pH values throughout the storage period, which was attributed to microbial activity and the rapid consumption of free sugar. Notable, pH levels was not significantly different (p > 0.05) among free RSHH sugar, RSHH-PLGA-NP, and RSHH + RSHH-PLGA-NP at all the storage points.

**Table 8:** pH changes in stirred yogurt samples during storage

Storage period (days)	Control	Free RSHH	RSHH-PLGA-NP	RSHH + RSHH-PLGA-NP
**Fresh**	4.50 ± 0.28	4.45 ± 0.12	4.51 ± 0.18	4.40 ± 0.11
**5**	4.42 ± 0.1	4.23 ± 0.11	4.44 ± 0.12	4.38 ± 0.2
**10**	4.40 ± 0.12	4.00 ± 0.15	4.37 ± 0.29	4.25 ± 0.1
**15**	4.33 ± 0.15	3.86 ± 0.2	4.25 ± 0.22	3.97 ± 0.11
**20**	4.10 ± 0.27	3.80 ± 0.23	4.22 ± 0.15	3.92 ± 0.22

Data expressed as a mean of three replicates.

### Sensory assessment of stirred yogurt samples

Sensory evaluations of the loaded samples are shown in Table 9. Fresh samples recorded high flavor scores, with the control sample scoring the highest, followed by samples containing RSHH-PLGA-NP. By day 20, flavor scores decreased across all treatments. The samples enriched with RSHH-PLGA-NP retained higher flavor scores compared to those with free RSHH.

**Table 9:** Sensory evaluation of stirred yogurt samples

Storage period (days)	Sensory parameters	Control	RSHH	RSHH-PLGA-NP	RSHH + RSHH-PLGA-NP
**Fresh**	**Flavor (50)**	50 ± 2	48 ± 1	49 ± 2	48 ± 2
**20**		45 ± 2.5	40 ± 1	42 ± 1	42 ± 1
**Fresh**	**Appearance and color (10)**	10 ± 1	8 ± 1	9 ± 1	8 ± 1
**20**		9 ± 1	7 ± 1	8 ± 1	7 ± 1
**Fresh**	**Body and texture (40)**	40 ± 2	40 ± 2	40 ± 2	40 ± 2
**20**		38 ± 2	37 ± 2	38 ± 1	37 ± 1

Data expressed as a mean of three replicates.

Appearance and color scores were initially high across all samples but decreased after 20 days of storage. Samples fortified with free RSHH showed the most significant reduction in appearance scores. Samples with RSHH-PLGA-NP retained higher scores, reflecting better stability during storage. Body and texture scores were similar for all fresh samples, with a maximum score of 40. After 20 days, scores decreased slightly across all treatments. Samples containing RSHH-PLGA-NP maintained relatively higher scores. Statistically, the difference were not significant different (*p* >0.05) among the treatments at different storage points.

### Reducing sugar content during storage

The reducing sugar content of the yogurt samples exhibited distinct patterns depending on the form of RSHH incorporated. The control sample, which did not contain any RSHH, showed no change in reducing sugar levels throughout the storage period. Similarly, yogurt fortified with RSHH-PLGA-NP retained similar reducing sugar levels from Day 0 to Day 20. In contrast, yogurt with free RSHH initially had a significantly higher reducing sugar content of 18.044 ± 0.723 mg/mL on Day 0. However, this value declined sharply over the storage period, reaching to similar concentration as both of control and RSHH-PLGA-NP by Day 15.

## Discussion

Rice is one of the most widely grown crops in the world that generates about 1.35 tons of rice straw byproducts each ton of harvested rice grain^32^. These generated byproducts are abundant and renewable source for various valuable compounds based on their constituents. Cellulose (38.3%), hemicellulose (31.6%) and lignin (11.8%) are their main constituents^33^. Hemicellulose is a complex carbohydrate polymer with a diverse group of heteropolysaccharides, including xylans, xyloglucans, mannans, glucomannans and others that can be explored in various biomedical and industrial applications. The aim of this work was to explore the potential of RSHH as a bioactive compound with anticancer and prebiotic properties, and to investigate its applicability in innovative synbiotic formulations. This included the concept of developing a synbiotic yogurt that combines immediate probiotic support with sustained, prebiotic delivery through entrapment in PLGA nanoparticles. This approach addresses the growing demand for functional foods with enhanced health benefits, offering a novel strategy for balancing short- and long-term gastrointestinal health benefits.

Hemicellulose-derived hydrolyzates, rich in oligosaccharides and monosaccharides, have gained attention for their potential applications in prebiotics and functional foods^34^. The extraction of hemicellulose from rice straw using a mild alkaline solution at 80 °C is a standard yet efficient approach^35^. The use of a strong base like sodium hydroxide is highly effective in dissolving hemicellulose and lignin under specific conditions, making it a valuable tool in biomass pretreatment^35^. One of the key benefits of the alkali process is its ability to target lignin for removal while minimizing damage to carbohydrates. In this study, the extracted hemicellulose fraction was furtherly hydrolyzed by xylanase in order to prepare water soluble RSHH. Although several thermo-chemical techniques have been applied in the hydrolysis of the extracted hemicelluloses, the use of enzymes is the preferred method in food and pharmaceutical applications^36^. Moreover, NaOH pretreatment plays a critical role in reducing the inherent recalcitrance of biomass. It achieves this by breaking the complex linkages between lignin and hemicellulose, degrading β-O-4 bonds, cleaving ester bonds, and partially depolymerizing the lignin structure. These chemical alterations significantly lower the resistance of biomass to enzymatic attack, facilitating more efficient conversion processes in bioproduct applications^35^. In this study, xylanase was employed for the hydrolysis of hemicellulose, achieving an enzyme-to-substrate ratio that proved highly effective. The process yielded 5 g of RSHH from every 10 g of hemicellulose, highlighting its efficiency.

Characterization of the RSHH highlights its composition and suitability for potential applications. The high total reducing sugar content indicates a significant presence of oligosaccharides, reflecting successful enzymatic hydrolysis. Additionally, the total phenolic content provides insight into the bioactive properties of the product, which may contribute to antioxidant characteristics^37,38^. Our findings confirmed that RSHH exerts cytotoxic effects in a dose-responsive manner, potentially disrupting cellular processes vital for the survival of EAC cells. However, we acknowledge that assessing anticancer activity based on a single cell line provides only preliminary evidence. While our results demonstrate a significant reduction in EAC cell viability, future studies will expand on these findings by evaluating the anticancer potential of RSHH against multiple cancer cell lines and incorporating additional mechanistic assays such as apoptosis detection, cell cycle analysis, and oxidative stress markers. These further investigations will help confirm whether the observed cytotoxic effects are specifically due to direct anticancer activity or influenced by other confounding factors. The anticancer activity could be attributed to the higher content of XOS in the RSHH. For instance, it was reported that β-1,3 xylooligosaccharides derived from green algae induced apoptosis and significantly reduced the viability of MCF-7 human breast cancer cells in a dose-dependent fashion^39^. Similarly, another study observed the antitumor activity of XOS through in vitro cytotoxicity assays, further supporting the potential therapeutic applications of XOS in cancer treatment^40^.

Beyond RSHH exhibited significant prebiotic activity, further emphasizing its versatility as a health-promoting agent. An ideal prebiotic should selectively enhance the growth of beneficial bacteria, such as *Lactobacillus* and *Bifidobacterium* species, without promoting the proliferation of pathogenic microorganisms^10^. This selectivity is crucial because it ensures that the prebiotic contributes to a healthy gut microbiome, supporting functions such as improved digestion, enhanced immune response, and protection against infections. Non-selective stimulation of bacterial growth could inadvertently encourage pathogens, potentially disrupting the gut microbiota and leading to adverse health effects. In this context, the observed selective prebiotic activity of RSHH in the presented study is particularly promising. Not only did RSHH improve the growth of beneficial bacteria, but it also managed to lower the growth of pathogenic microorganisms. This effect could be attributed to the natural antioxidants and phenolic compounds presented in the RSHH which efficiently reduce the biological activity of harmful microbes^41^. This dual action underscores RSHH’s potential as a selective and effective prebiotic. This finding aligns with the results of Pattarapisitporn et al., who showed that RSHH outperformed commercial XOS in supporting the growth of *L. brevis*, achieving population levels similar to those with glucose^42^. Chapla et al. further explored this by employing β-xylosidase-free xylanase to enzymatically derive sugars from rice straw. The prebiotic potential of these sugars was confirmed through in vitro fermentation with *Bifidobacterium* spp., showing enhanced growth across all lactic acid-producing bacteria^43^. Notably, exposure to rice straw-derived XOS resulted in growth stimulation ranging from moderate to significant, outperforming the effects observed with xylose and inulin, as highlighted by Jaichakan et al.^44^.

While the prebiotic activity of RSHH highlights it’s potential to promote beneficial gut microbiota, its safety and suitability for long-term use are equally critical. The in vivo biosafety study serves as a cornerstone in validating the practical applicability of RSHH for human consumption and potential therapeutic applications. By demonstrating the absence of adverse effects on critical physiological parameters, including body weight, organ health, and serum biochemistry, this study underscores the biocompatibility of RSHH. Specifically, the lack of significant changes in liver and kidney function markers, along with an improved lipid profile in treated animals, provides robust evidence of the safety of RSHH at the tested dosages^45^. However, we acknowledge that the study duration was relatively short to fully substantiate the long-term safety of RSHH. A more comprehensive evaluation incorporating extended exposure periods, additional biomarkers of toxicity, and histopathological analyses would be necessary to confirm its safety profile. Future studies will focus on these aspects to provide a more rigorous assessment of RSHH’s potential effects. Despite these limitations, the obtained safety results are completely in agreement with those reported by Boonchuay et al., that confirmed that the European Food Safety Authority (EFSA) has approved XOS as a novel food, and the Food and Drug Administration (FDA) in the United States has deemed it acceptable for use in foods, with no harmful effects at a single dose of 5 g/kg of weight in Wistar rats^46^. It is worth noting that XOS is the main oligosaccharide present in RSHH, contributing significantly to its prebiotic activity. Furthermore, the consistent weight gain in both control and treated groups, without significant differences, indicates that RSHH does not induce any toxic effects that might impair growth or metabolism. These findings are pivotal as they bridge the gap between the preclinical evaluation of bioactive compounds and their potential application in functional foods or therapeutic products.

Next, RSHH was entrapped into PLGA NPs. This was undertaken to prevent its premature consumption by probiotic bacteria and starter cultures during yogurt storage. Probiotics and starter cultures are metabolically active and can utilize free oligosaccharides as a nutrient source, potentially depleting the RSHH before it reaches the gastrointestinal tract. Entrapment provides a protective barrier around the RSHH, ensuring its preservation during storage and enabling its targeted release in the gut^3,6,7^. The characterization results of the nanoparticles (NPs) confirm the successful entrapment of RSHH within PLGA nanoparticles, achieving moderate entrapment efficiency. The double emulsion method was chosen for NP fabrication as it aligns well with the water solubility properties of RSHH. Nonetheless, a notable limitation of this method is the reduced entrapment efficiency, which arises from the tendency of water-soluble compounds to diffuse into the outer aqueous phase during the secondary emulsion process, explaining the observed moderate efficiency^6^. The nanoscale size and spherical morphology of the NPs support their suitability for targeted delivery applications. Additionally, the zeta potential measurements demonstrated colloidal stability, preventing particle aggregation and ensuring consistent performance in biological systems^6^. Further, the release of RSHH from PLGA nanoparticles was monitored by measuring the reducing sugar content in yogurt samples over the storage period. The data showed no significant increase in reducing sugar levels in the yogurt containing RSHH-PLGA nanoparticles, confirming that RSHH was not released. This stability can be attributed to the acidic pH of yogurt, which inhibits the degradation of PLGA nanoparticles, thereby preventing premature release of the encapsulated prebiotic. PLGA is known to degrade primarily under neutral or basic conditions, while its stability in acidic environments prevents premature release of the encapsulated prebiotic^26^. Similarly, recent studies have highlighted the effectiveness of nanoparticle-based systems in improving the stability and targeted delivery of prebiotics in functional foods. A pivotal study by Fayed et al.^6^ demonstrated the successful encapsulation of inulin in PLGA nanoparticles. In vitro release assays conducted in simulated gastrointestinal fluids revealed that the nanoparticles exhibited strong resistance to acidic degradation. Upon exposure to simulated intestinal fluid, a rapid burst release occurred followed by a sustained release lasting up to 3 days. In addition to PLGA-based systems, chitosan–pectin nanoparticles have also shown promise in protecting prebiotics from gastrointestinal breakdown. These nanoparticles, formed via polyelectrolyte complexation, offer tunable physicochemical properties. Li et al.^47^ reported that such systems displayed significant resistance to acidic environments and exhibited a pH-responsive release pattern—minimal release in gastric conditions and enhanced delivery under neutral to alkaline pH, simulating the intestinal milieu^47^. These attributes underscore the potential of nanoparticles as an effective delivery system for prebiotics, making them promising candidates for use in functional food or therapeutic applications.

Finally, the evaluation of stirred yogurt fortified with the probiotic *L. paraplantarum*, free RSHH, and RSHH-PLGA-NP demonstrated the successful formulation of a synbiotic product that combines immediate probiotic support with entrapped prebiotic available for long-term gastrointestinal health benefits. The free RSHH sample initially supported higher *L. paraplantarum* counts compared to the entrapped RSHH-PLGA-NP sample and the control, reflecting the rapid availability of free sugar to the probiotics. However, these counts declined significantly after 15 days, indicating that free RSHH was depleted during storage and would not be available to enrich the gastrointestinal microbiota post-consumption. In contrast, the sample containing only RSHH-PLGA-NP maintained lower probiotic counts throughout storage, suggesting that entrapped RSHH alone cannot adequately support probiotic growth during this period. Additionally, the lack of probiotic or starter culture growth in our study suggests that RSHH was not released from the nanoparticles, consistent with previously reported finding^48^. Additional evidence supporting this conclusion includes the observed pH levels of the yogurt samples. When RSHH-PLGA-NPs were added alone, the pH did not drop, indicating that no sugar was released from the nanoparticles and fermentation did not occur, while the faster pH decline in samples containing free RSHH is attributed to the rapid fermentation of free sugar by the probiotics and starter cultures^49^. Furthermore, measurements of reducing sugar in the samples showed that yogurt containing RSHH-PLGA-NPs had the same sugar content as the control and less sugar compared to samples containing free RSHH. These findings reinforce that entrapped RSHH within PLGA nanoparticles remains unavailable during storage, highlighting the need to combine free RSHH with entrapped RSHH to achieve optimal functionality. Sensory evaluation further supports the advantages of using RSHH-PLGA-NP. Samples containing entrapped RSHH retained higher scores for flavor, appearance, and texture over the storage period. These results suggest that the entrapment of RSHH in PLGA nanoparticles preserved the sensory qualities of the yogurt.

Despite the promising results, this study has several limitations. First, while PLGA is FDA-approved, the in vivo safety of RSHH-loaded nanoparticles was not evaluated and warrants further investigation. Second, the moderate entrapment efficiency observed using the double emulsion method may limit scalability, highlighting the need for optimization. Finally, although anticancer and antioxidant activities were observed in vitro, their mechanisms and in vivo relevance remain to be validated. Future research should address these aspects through mechanistic studies and extended safety assessments.

To conclude, this study presents a novel synbiotic yogurt fortified with RSHH in both free and nanoparticle-encapsulated forms. Free RSHH supported immediate probiotic viability, while encapsulated RSHH ensured long-term prebiotic delivery. This dual-delivery strategy enhanced yogurt functionality and shelf stability, offering a sustainable and effective approach for gut health support. The findings support further exploration of agro-waste-derived bioactives and nanoparticle carriers in functional food development. Future studies should focus on optimizing encapsulation efficiency, and exploring the broader applicability of this system in non-dairy food matrices.

## Methods

### Production of bioactive hydrolyzate from rice straw hemicellulose

The bioactive RSHH was produced as previously outlined by Ismail et al. ^31^. Initially, extraction of rice straw hemicellulose fraction was carried out as follow: rice straw was collected, cut into small pieces, ground, washed thoroughly under running tap water, and dried at 50 °C for 24 h. For extraction, a 100 µL of 4% NaOH solution was added to every 10 grams of rice straw. The mixture was then subjected to thermal treatment at 80 °C for 1 h and subsequently allowed to incubate for 24 h. The extracting solution was filtered using a cotton mesh and adjusted to pH 5 using 37% HCl. Hemicellulose was then precipitated by adding twice the volume of 95% ethanol and left to incubate for 24 h. The resulting precipitate was filtered, washed multiple times with 95% ethanol, air-dried, collected, and ground into a fine powder.

The hemicellulose fraction obtained was hydrolyzed using xylanase, produced by the fermentation of rice straw using *Aspergillus terreus* (Accession no. MN368221), with an enzyme-to-substrate ratio of 3.75 U/mg. The hydrolysis was carried out in a 0.05 M acetate buffer (pH 5) and incubated for 4 hours at 40 °C. After hydrolysis, the reaction mixture was boiled for 10 minutes to stop the enzyme’s catalytic activity. The mixture was then centrifuged at 1750 g and 4 °C for 10 min. The clear supernatant was air-dried, collected, and stored under freezing conditions (-20 °C) for further use.

The moisture and ash content of the dried sample were determined by gravimetric analysis^50^. The total reducing sugar content was measured using the Miller method^51^, with xylose as the standard, while the total phenolic content was assessed using the Folin–Ciocalteu method, with gallic acid as the standard^52^. The active functional groups of the dried sample were analyzed using an FTIR spectrophotometer (Vertex 80 v, Bruker, Berlin, Germany), and its sugar components were estimated by high performance liquid chromatography (HPLC) analysis (Agilent Technology 1100 series), employing a refractive index detector, a Shim-pack SCR-101N separating column, and ultra-pure water as the mobile phase at a flow rate of 0.7 mL/min.

### Bioactivity of RSHH

The antioxidant activity was estimated according to Brand-Williams method based on the scavenging of 2,2-diphenyl-1-picrylhydrazyl (DPPH)^53^. A reaction mixture (4 mL) containing 100 μL of 1% w/v sample solution mixed with methanolic solution of DPPH radical (1.1 × 10-4 mol/L) was observed for a decrease in absorbance at 515 nm after standing in the dark for 30 minutes. The antioxidant activity was expressed in µmole Trolox Equivalents (TE)/mg of the dry sample.

The cytotoxic activity was evaluated in vitro following the methodology outlined by Ahmed et al., with a minor modification, using the Trypan Blue cytotoxicity assay^54^. EAC cells were maintained in female Swiss albino mice through intraperitoneal inoculation with 2.0 × 10^6^ cells per mouse. The cells were then diluted in sterile physiological saline solution to achieve a concentration of 10^6^ cells/mL. A 0.1 mL aliquot of the diluted cells was incubated at 37 °C for 120 minutes with varying concentrations of the sample. After incubation, 100 μl of trypan blue dye (0.4% in PBS, Sigma-Aldrich, St. Louis, USA) was added. The number of viable cells was determined using a hemocytometer, where non-viable cells stained blue, and viable cells remained unstained. Tumor cell viability (%) was assessed following incubation with the samples (500,750, and 1000 μg/mL) and the standard drug Vincristine (100 μg/mL).

### Prebiotic activity of RSHH

In this study, various probiotic strains were examined, including *Lacticaseibacillus rhamnosus* (*L. rhamnosus*) NRRL B-442, *Lacticaseibacillus casei* (*L. casei*) NRRL B-1922, *Lactobacillus gasseri* (*L. gasseri*) NRRL B-14168, *L. paraplantarum*, *Lactobacillus acidophilus* NRRL B-4495, *Ligilactobacillus salivarius* (*L*. *salivarius*) NBIMCC 1589, *B. bifidum* NRRL B-41410, and *Bifidobacterium lactis* (*B. lactis*) Bb12, obtained from the Dairy Department, National Research Center. Several of the *Lactobacillus* strains were reclassified following the novel taxonomy proposed by Zheng et al. ^55^. Additionally, pathogenic strains such as *Staphylococcus aureus* (*S. aureus*) ATCC 6538, *Escherichia coli* (*E. coli*) ATCC 8739, *Listeria monocytogenes* (*L. monocytogenes*) ATCC 5980, *Salmonella typhimurium* (*S. typhimurium*) ATCC 14028, *Aspergillus flavus* (*A. flavus*) ATCC 9643, and *Candida albicans* (*C. albicans*) ATCC 10231 were also investigated.

### Probiotic enrichment assay of RSHH

The basal de Man, Rogosa, and Sharpe medium (MRS) broth medium was used for cultivating the probiotic strains, while nutrient broth medium was used for the pathogenic strains. Both media were separately supplemented with 2% (w/v) sample and xylose. Each medium was inoculated with 10^5^ to 10^6^ cfu/mL of the respective probiotic strains or pathogenic strains. The probiotic strains were incubated for 48 hours at 37 °C, while the pathogenic strains were incubated at 37 °C for 24 h. *Aspergillus flavus* and *Candida albicans* were incubated at 25 °C for 48 h. Inulin was used as a prebiotic standard at the same concentration, and normal MRS broth served as a growth control. At the end of the incubation period, viable cells in the culture media were counted using the pour plate method: MRS agar was used for probiotic strains, and nutrient agar for pathogenic strains. Probiotic plates were incubated at 37 °C for 48 h under anaerobic conditions, while pathogenic bacterial strains were incubated at 37 °C for 24 h. *Aspergillus flavus* and *Candida albicans* plates were incubated at 25 °C for 36 h^56^.

### In-vivo biosafety

Twelve Wistar male rats, weighing between 132 ± 4.51 and 154 ± 2.00 g on average, were employed in the investigation. These were raised in the National Research Center’s animal house in Giza, Egypt. The rats were split into two groups, with six rats in each group. They were kept in housing at 25 °C with a photoperiod of 12 hours of light and 12 hours of darkness. They were fed rodent pellets and water. All procedures were carried out according to the rules of the Ethics Committee of the National Research Centre.

The mice were dosed for 28 days, including the day the experiment ended. The body weight of each rat was measured at (day 0, 14, 28), All of the animals were euthanized using cervical dislocation (CD) under anesthesia or tranquilization through a bit of ether for a very little pain. Death in animals was confirmed by the absence of pulse and breathing. Blood samples were taken from each rat and placed in plastic test containers. For biochemical measurements, the clotted blood samples were frozen at -20 °C and clear serum samples were aspirated off after a 10-minute centrifugation at 1107 g. The testicles, liver, kidney, heart, lungs, spleen, and brain were meticulously removed and weighed.

For the determination of serum biochemical parameters, the procedures outlined in the instructions for the Biodiagnostic kit (Biodiagnostic Company, Dokki, Giza, Egypt) were followed. Serum Aspartate Aminotransferase (AST) and Alanine Aminotransferase (ALT) levels were assessed using the methods described by Young^57^ and Lopez-Virella et al.^58^. High Density Lipoprotein (HDL) cholesterol was measured following the procedures of Lopez-Virella et al.^58^, while Low-Density Lipoprotein (LDL) cholesterol was calculated using the method of Wieland and Seidel^59^. Total cholesterol levels were determined according to Richmond^60^, and triglycerides were measured using the method of Fassati and Prencipe^61^. Urea and creatinine concentrations were assessed based on the methods of Tobias et al.^62^.

### Preparation of PLGA nanoparticles loaded with RSHH (PLGA-NP-RSHH)

RSHH was entrapped in PLGA nanoparticles (RSHH-PLGA-NP) using a modified double emulsion/solvent evaporation technique^7,26^. An aqueous solution (0.5 mL) containing 10 mg/mL of the sample was combined with 25 mg of PLGA (Rosemer 503H, Sigma) dissolved in 2.5 mL of dichloromethane. The mixture was emulsified using a probe sonicator set at 40% power for 1 min. This primary emulsion was gradually added to 10 mL of an aqueous solution containing 2.5% polyvinyl alcohol and subjected to five additional rounds of probe sonication (1 minute per cycle) at the same power. The resulting double emulsion was stirred at 110 g for 2 hours at room temperature to facilitate the complete evaporation of the organic solvent. Nanoparticles were then recovered by centrifugation at 7000 g for 45 min, washed with distilled water, and stored at −20 °C for subsequent use.

### Characterization of the prepared nanoparticles

The entrapment efficiency was assessed indirectly by collecting a specific volume of the supernatant after nanoparticle separation. The unentrapped RSHH was quantified using the Miller method, with dinitrosalicylic acid (DNS) reagent^51^. The entrapment efficiency percentage was then calculated using the following formula:

1 $$\mathrm{Entrapment\; efficiency} \% =\left(\frac{\mathrm{Total\; amount\; of\; RSHH\; used}-\mathrm{amount\; unentrapped}}{\mathrm{Total\; amount\; of\; oligosaccharide\; used}}\right){\rm{X}}100$$

The effective diameter, size distribution, and zeta potential of the RSHH-PLGA-NP were measured using a Zetasizer (Malvern, Cambridge, UK). The nanoparticles were first diluted with distilled water, and their effective diameter and polydispersity index were analyzed via Dynamic Light Scattering (DLS) at 25 °C. The zeta potential (mV) was determined using laser Doppler velocimetry.

Samples for TEM analysis were prepared by placing a 2 µL drop of Quantum Dots-Polymer (QD-P) or Quantum Dots (QD) onto a cleaned plasma-treated thin holey carbon copper grid (400-mesh). After allowing the sample to sit for 30 min, the excess liquid was carefully removed using angled filter paper. The sample was stained with 1% phosphotungested acid then High-resolution TEM (HRTEM) images were captured using (JEM-2100 electron microscope; Jeol, Tokyo, Japan) with a lattice-fringe resolution of 0.14 nm at an accelerating voltage of 200 kV.

### Production of functional stirred yogurt

Pasteurized cow’s milk (containing 4.5% total protein, 3.4% fat, 5.9% lactose, and 78.9% water) was heated at 86 °C for 15 min to prepare stirred yogurt^63^. After cooling the milk to 42 °C, it was inoculated with 2% (w/w) yogurt cultures (*Lactobacillus bulgaricus* and *Streptococcus thermophilus*) and 2% (w/w) probiotic *L. paraplantarum*. The inoculated milk was divided into four groups: Control (C) was probiotic stirred yogurt, treatment 1 (T1) was probiotic stirred yogurt with 2% free RSHH, treatment 2 (T2) was probiotic stirred yogurt containing 2% PLGA-NP-RSHH, and treatment 3 (T3) was probiotic stirred yogurt with 2% free RSHH and 2% PLGA-NP-RSHH. Each sample was poured into 100 mL plastic cups and incubated at 42 °C for 3 to 4 h until coagulation. After incubation, the samples were refrigerated for one day, stirred with glass rods to produce a beverage-like yogurt, and evaluated for microbial and sensory properties over a 20-day period.

### Microbiological analysis of yogurt treatments

The pour plate method, along with 10-fold serial dilutions, was employed to assess the microbial load in all treatments. Appropriately diluted samples were plated to enumerate *L. paraplantarum* (using MRS agar supplemented with vancomycin (pH 5.6), and incubated aerobically at 30 °C for 48 h^64^. *Streptococcus thermophilus* counts were determined using M17 agar, incubated aerobically at 37 °C for 48 h. *Lactobacillus bulgaricus* was enumerated on MRS agar containing 10% sorbitol and incubated anaerobically at 37 °C for48 h ^64^. Microbial populations were expressed as log CFU/mL. Additionally, the pH values of the stirred yogurt treatments were monitored throughout storage.

### Sensory evaluation of yogurt treatments

The sensory evaluation of fresh stirred yogurt treatments was conducted to assess flavor (50 points), body and texture (40 points), and appearance and color (10 points). A 10-point hedonic scale (ranging from 1 = “dislike extremely” to 10 = “like extremely”) was used to rate the sensory attributes. A total of 25 trained panelists from the Dairy Department, National Research Centre, Giza, Egypt, participated in the evaluation. The panelists were selected based on prior experience in dairy product evaluation and were screened for sensory acuity and familiarity with yogurt products. The evaluation was performed in a well-lit, temperature-controlled sensory laboratory (25 ± 2 °C) designed to minimize external influences. Samples were coded with random three-digit numbers and presented in a randomized order to prevent positional bias. Each panelist received the samples in identical containers at 4 ± 1 °C under neutral white lighting. Unsalted crackers and water were provided to cleanse the palate between samples. The sensory evaluation procedure followed the methodology described by Mohamed et al., with necessary modifications to suit the study’s objectives^65^.

### Reducing sugar measurement of yogurt treatments

The reducing sugar content was analyzed in yogurt samples under different conditions (Control, Free RSHH, and RSHH-PLGA-NP). Measurements were performed at three time points: immediately after preparation (Day 0) at day 15 and at the end of the 20-day storage period. The reducing sugar concentration was determined using the Miller method, with dinitrosalicylic acid (DNS) reagent^66^. The yogurt was stored at 4 °C throughout the study. Samples were taken aseptically, diluted appropriately, and subjected to DNS assay. The absorbance was measured at 540 nm using a UV-Vis spectrophotometer, and the reducing sugar concentration was calculated using a standard curve of known RSHH concentrations.

### Statistical analysis

All experiments were conducted in triplicate, and data are presented as mean ± standard deviation (SD). Statistical analyses were analyzed by GraphPad Prism 8.00 for windows (GraphPad Inc., La Jolla, CA, USA). The data were processed by student t-test or one-way analysis of variance (ANOVA) using Tukey’s Multiple Comparison Test. A *P* value < 0.05 was considered as significant

## Data Availability

No datasets were generated or analysed during the current study.
